# Corneal myxoma following micro-pulse cyclophotocoagulation in a young female: A case report

**DOI:** 10.1016/j.ijscr.2024.109677

**Published:** 2024-04-21

**Authors:** Khalid M. Alshomar, Hind M. Alkatan, Hussam M. Al-Razqan, Ahmed S. Al-Saleh, Nouf A. Alzendi

**Affiliations:** a Ad Diriyah Hospital, Riyadh Third Health Cluster (Ministry of Health), Ad Diriyah, Saudi Arabia; b Department of Ophthalmology, College of Medicine, King Saud University, Riyadh, Saudi Arabia; c King Saud University Medical City, King Saud University, Riyadh, Saudi Arabia; d Department of Pathology and Laboratory Medicine, College of Medicine, King Saud University, Riyadh, Saudi Arabia; e Anterior Segment Division, King Khaled Eye Specialist Hospital, Riyadh, Saudi Arabia; f Glaucoma Division, King Khaled Eye Specialist Hospital, Riyadh, Saudi Arabia

**Keywords:** Corneal myxoma, Cyclophotocoagulation, Glaucoma

## Abstract

**Introduction:**

Corneal myxoma is a rare benign tumor that can occur in the cornea where the exact cause remains unknown. However, it is thought to be a reactive process that can be caused by corneal infections, ectasia, ocular trauma, or surgery.

**Presentation of case:**

In this case report, we describe a 35-year-old-woman who presented with corneal myxoma after micro-pulse cyclophotocoagulation (MPCPC). The patient presented with decreased vision due to a large pedunculated white corneal mass after being treated with MPCPC as a non-surgical management of her pre-existing glaucoma. The corneal mass was localized to the sub-epithelial space and was excised successfully by a minimal invasive procedure without recurrence over a 1-year period.

**Discussion:**

Corneal myxomas are unusual benign tumors typically seen in adults as whitish gelatinous lesion. Only a few individual cases and case series have been reported in the literature. The exact pathogenesis is yet to be known. The lesion is thought to be due to an inflammatory process. We are reporting a case of corneal myxoma that has occurred after MPCPC which is a non-surgical cyclodestructive procedure. The procedure has not been mentioned previously as a risk factor for corneal myxoma. Our case is the first corneal myxoma developing after MPCPC.

**Conclusion:**

We report a corneal myxoma noted in a young female after a non-surgical laser procedure. Though the lesion is rare, it should be considered in physicians' differential of a corneal mass especially in the presence of chronic ocular.

## Highlights

• Corneal myxoma is a rare benign tumor.
• Secondary corneal myxoma develop as reactive process noted in infections, ectasia and trauma or surgeries.
• The association of corneal myxoma and micro-pulse cyclophotocoagulation has not been previously reported in the literature.

## 1 Introduction

Myxomas are rare, benign lesions that originate from connective tissue and have been sporadically identified in various ocular structures including the orbit, eyelids, conjunctiva, and cornea [1,2]. The occurrence of the lesion in the cornea is particularly uncommon with cases being documented infrequently in the literature [1]. Corneal myxoma can be classified as primary corneal myxoma, which is extremely rare, and secondary corneal myxoma.

Secondary corneal myxomas usually develop as a reactive process noted in corneal infections, ectasia and after ocular trauma or surgeries [[3], [4], [5]]. To the best of our knowledge, micro-pulse cyclophotocoagulation (MPCPC) has not been previously mentioned in the English literature as a presumed inducing cause of corneal myxoma. Herein, we report the case of 36-year-old female presented with primary sub- epithelial corneal myxoma 1 year after MP CPC. This case report has been reported in accordance with the SCARE criteria [6].

## 2 Case description

**Fig. 1:**
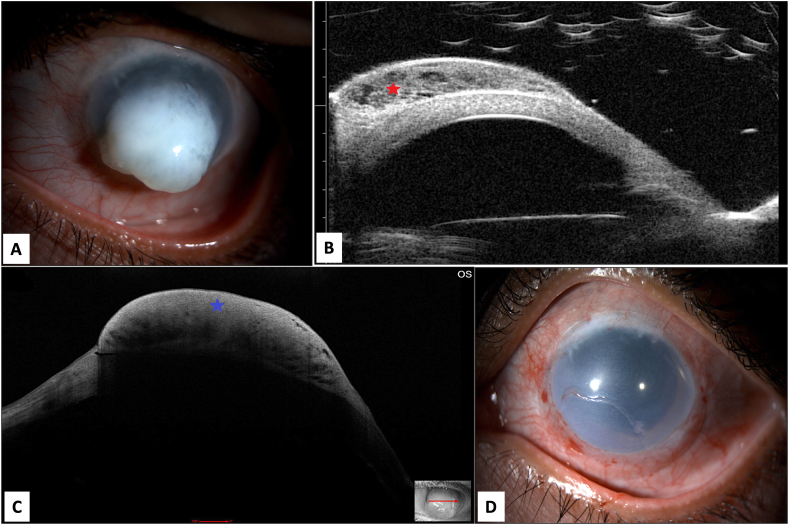
A. The clinical appearance of the protruding whitish gelatinous left corneal mass. B: The UBM showing a cystic mass over the cornea localized to the anterior space without invasion. (Red star) C: The AS-OCT shows a hyper-reflective lesion within the sub-epithelial space with cystic space. (Blue star) D: The appearance of the left cornea following excision of the lesion.

**Fig. 2:**
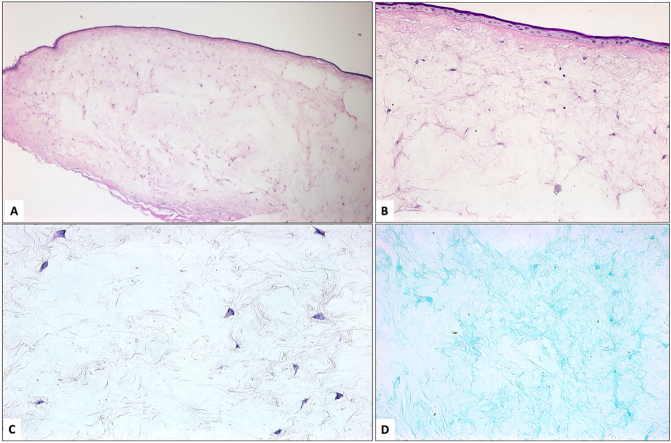
A: Histopathological appearance of the left corneal lesion with thin epithelium, absent Bowman's layer, and hypocellular subepithelial myxoma (original magnification x40 Periodic acid Schiff). B: Higher power of the same findings in the corneal myxoma with stellate and spindle-shaped cells (original magnification x200 Periodic acid Schiff). C: The stellate and spindle-shaped cells within loose stroma (original magnification x400 Hematoxylin and eosin). D: Alcian blue-rich stroma of the lesion (original magnification x200 Alcian blue).

A 35-year-old lady, not known to have systemic illnesses, presented to the emergency department (ED) at our institute complaining of pain in her left eye. The patient had a history of retinal surgery done at age of 5 years following blunt trauma with post- traumatic dislocated crystalline lens. The patient underwent a secondary intraocular lens (IOL) implantation in the affected eye as planned shortly after her retinal surgery. Fifteen years later, at the age of 20, trabeculectomy was performed at a different center and remained stable for about 9 years. Upon presentation to our hospital, the patient was using brimonidine 0.15 % twice daily, dorzolamide 2 % twice daily and timolol 0.5 % twice daily eye drops. Her vision in the affected left eye was hand motion with 38 mmHg intraocular pressure (IOP) that responded to stat medications including brimonidine 0.15 %, dorzolamide 2 % and timolol 0.5 % given in our ED and dropped to 10 mmHg. The slit lamp examination of the left eye showed a flat bleb, decompensated cornea with significant corneal edema, mid-dilated pupil, mild dislocated IOL and advanced optic nerve cup to disc ratio. The patient was discharged from the ED on full topical antiglaucoma agents in addition to oral acetazolamide and was seen after 2 weeks in the outpatient department (OPD). The patient was compliant on her drops, yet her IOP measured 40 mmHg at the time. She was booked for micro pulse- cyclophotocoagulation (MP-CPC) under local anesthesia. The procedure was conducted with MP3 micro-pulse probe (IRIDEX Inc.) where it was placed at the limbus with the notch facing the limbal side and the probe was perpendicular to the surface of the globe. The laser settings were 2000 mW power with a duty cycle of 31.33 % treating 360 degrees sparing three and nine O'clock areas. Each hemisphere is treated with 5 passes over 90 s. After the procedure, prednisolone acetate 1 % initiated every 2 h and tapered weekly over 6 weeks along with Atropine 1 % twice a day and prophylactic ofloxacin 4 times a day for one week. The IOP was controlled postoperatively with the same baseline topical medication, and she was followed up in OPD regularly. In her 1-year follow up visit the patient presented with pain and inability to close her left eyelids fully due to a protruded white corneal mass which has been increasing in size with time. The vision of the left eye was only light perception with good projection and her IOP was estimated to be 30–40 mmHg digitally. On examination, she had a large pedunculated whitish corneal mass (Fig. 1A). Ultrasound Biomicroscopy (UBM) showed a cystic mass over the cornea localized to the anterior space without invasion and the anterior segment Optical Coherence Tomography (AS- OCT) revealed a hyper-reflective lesion within the sub-epithelial space with cystic space (Fig. 1B & C). Initially, the provisional diagnoses were either corneal keloid or acquired corneal sub-epithelial hypertrophy. To reach definitive diagnosis, the patient was counseled to undergo surgical excision for therapeutic and diagnostic purposes. The patient was referred to a cornea specialist and after obtaining the patient's consent, the procedure was carried out in the minor treatment room under topical anesthesia. After sterilization and surgical preparation, the edge of the corneal lesion was found to be adherent to the underlying tissue. A crescent blade was used to dissect the edge to the resistance-free level. After releasing the edge, the whole lesion was successfully removed in one piece by peeling it using toothed forceps. It was found to be confined to the subepithelial space and separated from the underlying stromal bed in the center (Video 1). After full excision, a bandage contact lens was applied (Fig. 1D) and the patient received prophylactic topical ofloxacin 0.3 % eye drops four times a day for one week and topical ophthalmic prednisolone acetate 1 % eye drops in a tapering dose for one month. Histopathological examination of the excised tissue showed irregular thin elevated corneal epithelium with bullous changes at the periphery of the lesion (Fig. 2A). Bowman's layer was absent and the subepithelial lesion consisted of stellate-shaped and spindle cells within loose myxomatous background that is rich in glycosaminoglycans that was stained using Alcian blue (Fig. 2B, C & D). The tissue diagnosis was consistent with a corneal myxoma. The patient was seen on the first postoperative day and one week following the procedure. Visual acuity, intraocular pressure, and the corneal epithelial defect at the bed of the excised lesion were checked at follow-up. The patient showed total healing of the corneal epithelial defect after one week and did not develop recurrence over 6 months of follow-up. Later, she asked for cosmetic options and eventually underwent corneal tattooing with regular follow up visits to our OPD and a stable course.

## 3 Discussion

Corneal myxomas are rare tumors with a benign clinical course. Few individual cases and case series have been reported in the literature [1,4,5,8]. The exact pathogenesis for developing this lesion is controversial and yet to be known. Nevertheless, secondary lesions have been advocated to represent a reactive process seen in association with keratitis, corneal ulcers, keratoconus, Peter's anomaly, ocular trauma, and surgeries [1,[3], [4], [5]]. However, primary spontaneous lesions without any inducing reason have also been reported [1,[3], [4], [5]]. As suggested in the literature secondary corneal myxomas are due to inflammatory processes. Our patient underwent Micro-pulse cyclophotocoagulation (MPCPC) which is a non- surgical cyclodestructive procedure where diode laser is delivered via trans-scleral approach. However, none of the previously reported corneal myxoma cases developed in association with or following MPCPC in the English literature. We postulate the pathogenesis in our case to be a reactive response in a long-standing decompensated cornea that was provoked by the inflammatory process induced by the recent MPCPC procedure. The clinical manifestation in our case was similar to previously reported cases where adult patients usually present with whitish gelatinous lesion over the cornea [1,4,5,8]. The differential diagnoses in these cases include corneal scarring, Salzmann nodular degeneration, corneal keloid, and corneal squamous cell carcinoma [1,4,5,8]. To reach definitive diagnosis of the lesion excisional biopsy is often required; and histologically, myxomas are characterized by hypocellular and hypovascular stroma with abundant glycosaminoglycans rich in hyaluronic acid [1]. The cells lack pleomorphism and differentiate into modified fibroblasts and myofibroblasts with secretory activity. The primary skin lesions are more significant clinically and may be associated with Carney's complex. Similarly, corneal myxoma shows spindle-shaped and/or stellate cells within a loose stroma with transformation of stromal keratocytes into cells with myofibroblastic differentiation and prominent secretory activity leading to deep staining with Alcian blue [1]. They are thought to represent mostly a degenerative or a reactive process and lesions are typically located in the anterior cornea subepithelially disrupting the Bowman's layer with various extension into the stoma [1,5]. The management of corneal myxoma is mainly local excision. However, the surgical technique varies depending on the extent and exact location of the lesion within the corneal layers. Different surgical procedures have been mentioned obtaining the specimen and treating the patients such as superficial or lamellar keratectomy and keratoplasty [1,5,7]. In our patient, the lesion was noted to be confined to the subepithelial space and was successfully excised by a minimally invasive procedure with no recurrence over a period of 1 year.

## 4 Conclusion

Corneal myxomas are benign lesions that should be brought to ophthalmologists' attention. The lesions presents either as primary or secondary lesion. Secondary lesions are usually acquired as reactive processes to corneal infections, ectasia, ocular trauma, or following surgery. The association of corneal myxomas with trans-scleral laser procedures has not been previously reported in the literature. We report a case that highlights a unique presentation of the lesion where it occurred following a micro-pulse cyclophotocoagulation. This case serves as an addition to the limited reports of corneal myxoma in the literature emphasizing the need for further research into the causative factors.

The following is the supplementary data related to this article.

Supplemental Digital Content 1 Video illustrating the surgical technique of excising the lesion. Supplemental Digital Content 1

## Ethical approval

Ethical approval for this study (RD/26001/IRB/0078-23) was approved by the IRB department at King Khaled Eye Specialist Hospital, Riyadh, Saudi Arabia on 12/3/2023.

## Funding

No funding was needed for this case report.

## Author contribution

Author 1

Study Design, data collection & interpretation, writing the paper.

Author 2

Data collection & interpretation, writing the paper.

Author 3

Surgeon, data collection & interpretation, writing the paper.

Author 4

Surgeon, data collection & interpretation.

Author 5

Surgeon, data collection & interpretation.

## Guarantor

King Khaled Eye Specialist Hospital, Riyadh, Saudi Arabia.

## Registration of research studies

Not applicable.

## Consent

Written informed consent was obtained from the patient for publication of this case report and accompanying images. A copy of the written consent is available for review by the Editor-in-Chief of this journal on request.

## Declaration of competing interest

No conflict of interest.
